# The curious case of Gαs gain-of-function in neoplasia

**DOI:** 10.1186/s12885-018-4133-z

**Published:** 2018-03-15

**Authors:** Giulio Innamorati, Thomas M. Wilkie, Havish S. Kantheti, Maria Teresa Valenti, Luca Dalle Carbonare, Luca Giacomello, Marco Parenti, Davide Melisi, Claudio Bassi

**Affiliations:** 1 Department of Surgical Sciences, Dentistry, Gynecology and Pediatrics, University of Verona, Verona, Italy; 2 Pharmacology Department, UT Southwestern Medical Center, Dallas, TX USA; 3 Department of Medicine, University of Verona and Azienda Ospedaliera Universitaria Integrata Verona, Verona, Italy; 4 Department of Medicine and Surgery, University of Milano-Bicocca, Monza, Italy; 5 Laboratory of Oncology and Molecular Therapy, Department of Medicine, University of Verona, Verona, Italy

**Keywords:** GNAS, Heterotrimeric Gs protein, Activating mutation, Neoplasm, McCune Albright Syndrome, Intraductal papillary mucinous neoplasm, Fibrous dysplasia

## Abstract

**Background:**

Mutations activating the α subunit of heterotrimeric Gs protein are associated with a number of highly specific pathological molecular phenotypes. One of the best characterized is the McCune Albright syndrome. The disease presents with an increased incidence of neoplasias in specific tissues.

**Main body:**

A similar repertoire of neoplasms can develop whether mutations occur spontaneously in somatic tissues during fetal development or after birth.

Glands are the most “permissive” tissues, recently found to include the entire gastrointestinal tract. High frequency of activating Gαs mutations is associated with precise diagnoses (e.g., IPMN, Pyloric gland adenoma, pituitary toxic adenoma). Typically, most neoplastic lesions, from thyroid to pancreas, remain well differentiated but may be a precursor to aggressive cancer.

**Conclusions:**

Here we propose the possibility that gain-of-function mutations of Gαs interfere with signals in the microenvironment of permissive tissues and lead to a transversal neoplastic phenotype.

## Background

Heterotrimeric Gαβγ proteins are central to sensing a great number of extracellular stimuli. Each of the subunits is encoded by a multigene family that accommodates coupling to a huge diversity of seven transmembrane receptors. In metazoan organisms, four classes of Gα subunits, Gs, Gi, Gq and G12, couple several hundreds receptors to distinct sets of effector proteins. The Gs class of alpha subunits includes two genes GNAS and GNAL encoding Gαs and Gαolf proteins respectively that stimulate the effector protein adenylyl cyclase and regulate certain ion channels.

**Fig. 1:**
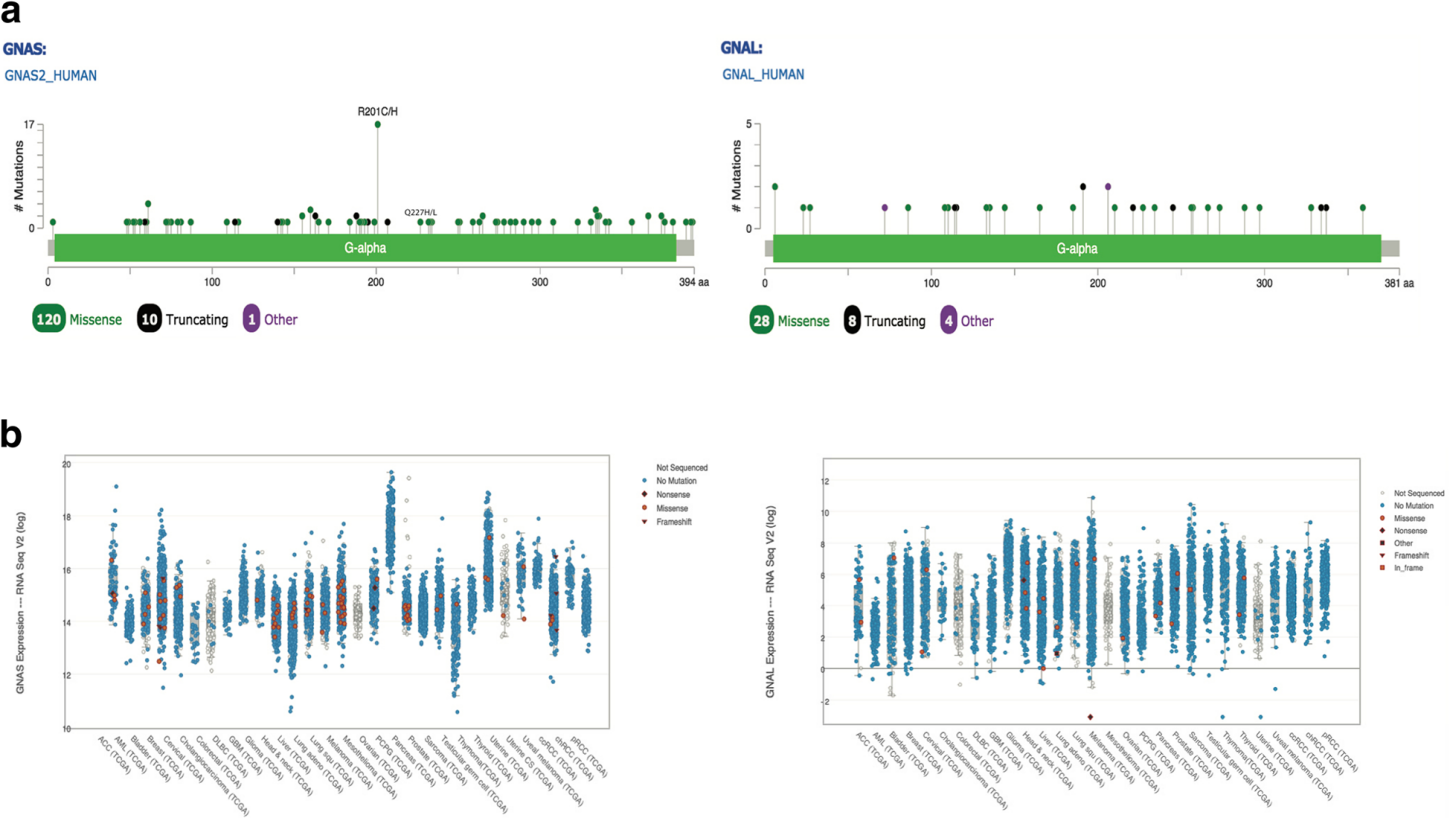
GNAS/GNAL Cross Cancer Comparison in Human Tumor Samples based on TCGA provisional data. Cbioportal was used to generate figures. **a** Mutational Diagrams. No activating alleles were found in GNAL. Two activating alleles were found in GNAS producing residues substitutions at R201 and Q227 (see in the text). **b** Expression Level Diagrams. GNAS shows higher expression level compared to GNAL in a cross cancer comparison

**Fig. 2:**
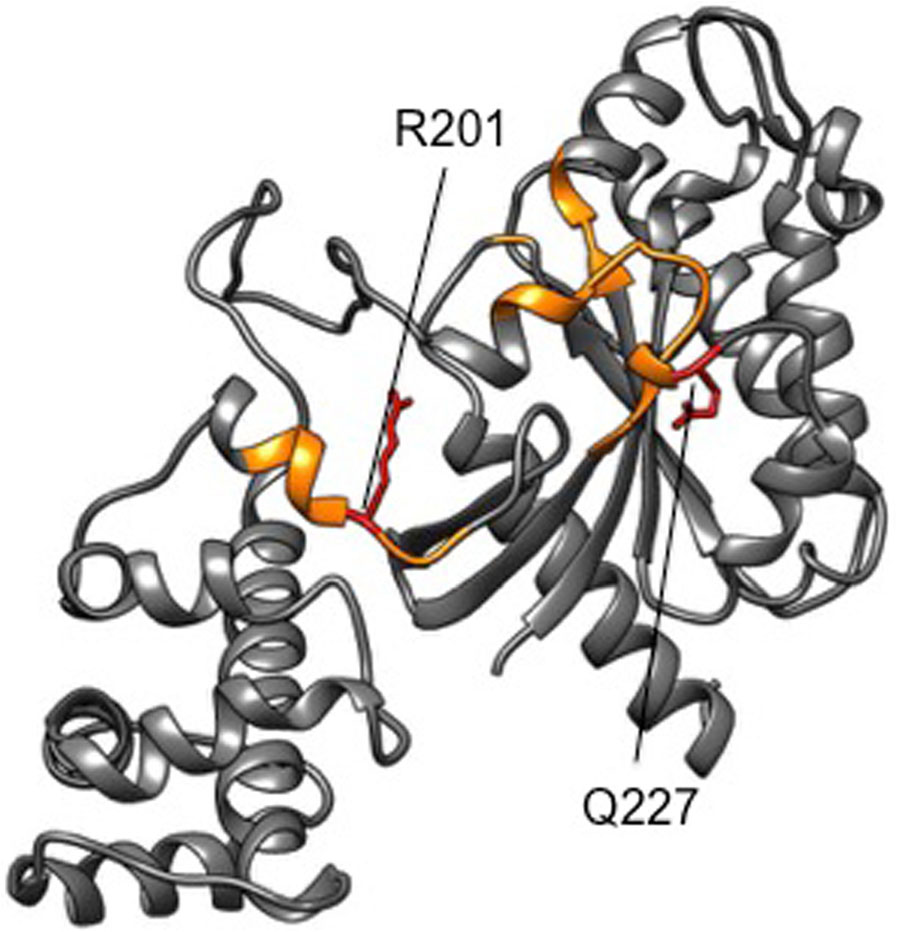
3-D model of GαS. Residues 40 to 394 of Gαs are represented based on protein model portal, template 3sn6A. The GTP binding domains are indicated in yellow, the most common *gsp* mutations, at R201 (the target of Cholera toxin) and Q227, are indicated in red

Whole genome analysis revealed that mutations affecting G proteins and GPCRs are more frequent than previously thought in transformed cells [1]. In a growing number of neoplasias, two hotspots on Gαs, Arg (R)201 and Gln (Q)227, are found mutated to three (Cys/His/Ser) or two (Arg/Leu) amino acids, respectively (Fig. 1a). By contrast, activating mutations in Gαolf have not been found. In Gαs, R201 mutations are more common than Q227 but substitutions at either residue inhibit intrinsic GTPase catalytic activity. The crystal structure shows these residues contribute to transition state interactions during GTP hydrolysis [2] (Fig. 2). As a consequence, mutant Gαs with substitutions at either residue remains GTP-bound, persisting in an active state that prolongs the effector protein interaction with dissociated Gαs or βγ. Cholera toxin achieves an equivalent result by ADP ribosylating R201 [3].

The TCGA database shows tissue distribution of Gαs activating mutations among 29 cancers (Table 1, Fig. 1b). An obvious indication for a cancer subtype is not evident. However, recent data point to certain highly prevalent neoplasms not included in the TCGA database (Table 2 and reference therein). The frequency of Gαs activating mutations in adenocarcinoma is comparable between the two data sets. The striking difference is the common occurrence of Gαs activating mutations in adenoma and other early neoplasia in neuroendocrine tumors. (Table 2). Gαs is associated with neoplasia and cancer but not Gαolf, probably because Gαs is ubiquitously expressed at high levels whereas Gαolf is highly expressed only in olfactory epithelium and other specialized cells (Fig. 1), and tumors from those tissues have not been well characterized. These features of effector protein regulation and expression pattern influence the oncogenic potential of Gs class genes.

**Table 1:** Activating alleles of Gαs and/or Rαs in cBioPortal cancer cohorts

Cancer Study Type	GNAS^b^	(%^c^)	GNAS^a,b^ RAS^b^	RAS^b^	Percent	Total Samples
AML	0		0	21	(11%)	191
Bladder	0		0	0	(0%)	131
Breast Cohort 1	1		0	6	(1%)	816
Breast Cohort 2	1		0	3	(1%)	482
Renal Clear Cell Carcinoma	0		0	0	(0%)	499
Colorectal Adenocarcinoma	1		0	91	(43%)	212
Head and Neck Squamous Cell	0		0	0	(0%)	279
Diffuse Glioma	0		0	8	(1%)	794
Lung Adenocarcinoma	2	(2%)	2	76	(33%)	230
Lung Squamous Cell Carcinoma	0		0	0	(0%)	178
Pan-Lung Cancer	5	(<1%)	3	232	(20%)	1144
High Grade Serous Ovarian Cancer	0		0	4	(1%)	316
Pancreatic Adenocarcinoma	7	(5%)	3	138	(93%)	149
Prostate Adenocarcinoma	0		0	0	(0%)	333
Stomach Adenocarcinoma	3	(1%)	1	28	(10%)	287
Papillary Thyroid Cancer	0		0	0	(0%)	507
Uterine Endometrial Carcinoma	0		0	51	(21%)	240

^a^Coincidence of GNAS and KRAS activating mutations. Among the cancer cohorts from TCGA in cBioPortal [100, 101], pancreatic adenocarcinoma, pan-lung, and stomach cancers had the highest frequencies of activating alleles of Gαs in the absence of KRAS mutations

^b^Activating alleles of GNAS and/or KRAS

^c^% GNAS activating alleles with two or more occurrences in the cancer cohort

**Table 2:** Incidence of “*gsp*” in “receptive organs”

Site	Histology (Dysplasia/Neoplasia)	Incidence % (n)	Refs
Thyroid	Toxic thyroid adenoma	**23% (65**)	[102–104]
	Non functional adenoma	0% (31)	[102, 104]
	Carcinoma	0% (18)	[104, 105]
Pituitary	GH-secreting adenoma	**41% (504)**	[106–126]
	Prolactin-secreting adenoma	0% (7)	[107]
	Non functioning	3% (32)	[107]
Muscle	Intramuscolar myxoma	**45% (101)**	[39, 127, 128]
	Various myxoid lesions	0% (105)	[39, 127–130]
Bone	Fibrous dysplasia (FD)	**81% (414)**	[127, 131–134]
	Low grade periosteal osteosarcoma	0% (11)	[135]
	Low grade central osteosarcoma	3% (35)	[135–137]
	Low grade parosteal osteosarcoma	0% (80)	[135, 137]
	Osteofibrous dysplasia	0% (13)	[132, 134, 138]
	Ossifying fibroma	0% (66)	[131, 132]
Blood	Hematological conditions	0.6% (512)	[139–141]
Kidney	Renal cell carcinoma	**17% (30)**	[142]
Lung	Mucinous cystoadenoma	0.5% (208)	[24, 68, 143]
Pancreas	Intraduct. Papill. Mucin. Neop.(IPMN)	**58% (809)**	[17, 19–21, 30, 68, 144–148]
	Incipient IPMN	**33% (21)**	[149]
	Intraduct. Tubulopapill. Neop. (ITPN)	**60% (15)**	[146]
	Intraepithelial neoplasia (PanIN)	2% (246)	[20, 150]
	Serous cystoadenoma (SCN)	0% (54)	[20, 144]
	Mucin. Cyst.Neop. (MCN)	0% (31)	[20, 144]
	Neuroendocrine tumor	0% (52)	[20]
	Ductal adenocarcinoma (PDAC)	0.4% (253)	[20, 30, 144, 145, 150]
Biliary tract	IPMN of the bile duct	**23% (120)**	[42, 151–153]
	Biliary intraepithelial neoplasia	1% (82)	[153, 154]
Stomach	Pyloric gland adenoma	**48% (23)**	[41]
	Gastric adenocarc. of the fundic gland	**24% (29)**	[64, 74]
	Hyperplastic gastric polyps	0% (10)	[127]
	Foveolar type adenoma	0% (23)	[41]
	Intestinal type adenoma	0% (34)	[41]
	Gastric adenocarcinoma	0% (71)	[41]
Duodenum	Pyloric gland adenoma	**92% (35)**	[41]
	Gastric foveolar metaplasia	**41% (66)**	[66]
	Gastric heterotopia	**28% (81)**	[66]
	Adenocarcinoma	**17% (30)**	[66]
	Gastroent. neuroen. tum. (GEP-NET)	0% (31)	[155]
Colon-rectum	Villous adenoma	**67% (55)**	[155–158]
	Tubular villous adenoma	4% (154)	[155–157]
	Tubular adenoma	0% (28)	[155, 157]
	Polyps	0% (45)	[156]
	Adenocarcinoma	3% (820)	[68, 155–159]
Liver	Normal liver Intrahepat.cholangioc	**12%(43)**	[160]
	Advanc. Intrahepatic cholangiocarc	3% (38)	[160]
	Hepatocell. Adenoma	4% (179)	[22, 161]
	Hepatocell. Carcinoma	0.8% (245)	[22]
	Fluke-ass. cholangiocarcinoma	9.3% (53)	[23]
Appendix	Low grade app. muc. neop. (LAMN)	**43% (84)**	[31, 40, 68, 162, 163]
	Adenocarcinoma	**46% (106)**	[31, 40, 68, 69, 162–164]
Gonads	Leydig cell stromal tumor	**67% (6)**	[165]
	Lobular Endocer. Glandular Hyperpl.	**28% (32)**	[166]
	Juvenile Ovarian granul. cell tumor	**30% (30)**	[167]
	Mucinous cystoadenoma	9% (45)	[68, 168]
	Mucinous border line tumor	4% (53)	[68, 168]
	Mucinous cystadenocarcinoma	2% (45)	[68, 168]
	Ovarian granulosa cell tumor	0% (25)	[169, 170]
	Other sex cord stromal tumors	0% (6)	[170]
	Adenocarcinoma	4% (92)	[166]
	Squamous cell carcinoma	0% (43)	[166]
Adrenocortical	Cortisol producing adenoma	**20% (25)**	[28, 90, 171]
	Adrenocrotical Carcinoma	3% (40)	[80]

An extensive list of neoplasias, flanked by *gsp* prevalence. Numbers in bold correspond to tumors presenting a rate over 10%. In each responsive tissue/organ, incidences in the double digits often pinpoint a single diagnosis that stands out among other virtually *gsp* negative tumor types. The large majority of other neoplasms are negative, for instance ref [50] analyzed 1126 cases falling within 15 diagnosis and found all negatives

Equivalent activating mutations of the Gq class proteins Gαq and Gα11 occur in virtually all blue naevi [4], and for Gαi2, in ovary and adrenal gland [5]. These activating mutations in Gα genes are analogous to oncogenic mutations in the small G protein Ras which inhibit GTP hydrolysis [6], present in about 30% of all tumors, reaching nearly 100% in specific types [7]. Such specific mutation profiles in G alpha genes, tightly linked to gain-of-function in benign neoplasia and some aggressive cancers, leaves little doubt about the selective advantage provided by the mutation to transforming cells. Yet, how this molecular mechanism contributes to the pathogenesis remains obscure.

## Main body

### GNAS and the activating mutation

Gαs is encoded by the *GNAS* locus. This is a highly complex gene with alternative promoters controlling the expression of multiple transcripts. Gαs is also regulated by genomic imprinting. In a few specific tissues, including the renal proximal tubule, the thyroid, the pituitary, and the gonads, one of the two alleles, usually the father’s, is silenced by methylation during development [8, 9].

In humans, germline transmission of Gαs activating mutations is not observed, suggesting they are embryonic lethal. However, postzygotic activating mutations are maintained through somatic mosaicism, causing McCune Albright Syndrome (MAS, MIM#174800). Predominant MAS symptoms are related to endocrine tumors (principally in pituitary, ovarian, adrenal and thyroid glands), skin pigmentation (cafè au lait) and polyostotic fibrous dysplasia.

When somatic mutations occur during adult life, the phenotype emerges independently in the same “permissive” tissues affected in MAS and displays analogous symptoms to postzygotic mosaicism. Over a quarter century ago, a direct link between *GNAS* gain-of-function mutations and cell transformation was established in growth hormone (GH)-secreting pituitary adenoma of acromegalic patients and in small subsets of other endocrine tumors [6, 10]. The constitutively active forms of GNAS (Fig. 2), generated by the hotspot mutations described in the previous paragraph, were collectively named *gsp* in recognition of their oncogenic potential.

### *Gsp*, the microenvironment and precursor cell maturation

The hypothalamic hypophysiotropic hormone (GHRH) is the principal regulator of secretory and proliferative functions of somatotrophs [11]. Gαs directly couples the GHRH receptor to its downstream effector proteins. This fully explains the secretory properties of pituitary adenoma that are common to most *gsp*+ tumors discovered, including exocrine (mostly mucous) and endocrine subtypes. The last category could be extended to dysplasia involving the bone marrow, as it produces signals that regulate other organs in the body (i.e. osteocalcin targeting pancreas and testis or fibroblast growth factor-23 (FGF-23) targeting kidney [12]).

In fibrous dysplasia (FD) *gsp* prevalence is over 80% (Table 2 at the bottom). Patients affected by this uncommon bone disorder present fibrous tissue in place of normal bone with lesions reflecting the dysfunction of osteogenic progenitors. The protein product of *gsp* indirectly promotes transcription of the proto-oncogene c-fos specifically in the marrow of affected bones [13]. In addition, the expression of osteoblast-specific genes is inhibited while IL-6 expression is increased, thus promoting the action of osteoclasts [14]. As a result, unorganized and poorly mineralized woven bone is observed with the retraction of osteogenic cells from the bone surface and the formation of Sharpey’s fibers. Hematopoietic marrow is replaced by fibrotic marrow with characteristics of osteogenic progenitors that hyperproliferate but cannot completely differentiate into osteoblasts. Surprisingly, the mutation introduced in a murine zygote was transmissible. It did not produce endocrine neoplasm but did replicate human fibrous dysplasia [15]. In this animal model, the expression of mutant Gαs was achieved by viral transduction thus leaving the natural *GNAS* locus unaffected. Despite this bias, the experiment proved that functional up-regulation of Gαs, per se, preserves most functional aspects specific of stem cells or of osteoblastic progenitors but it compromises cell organization in the tissue.

In summary, by conditioning stem cell maturation, *gsp* dramatically compromises the hematopoietic microenvironment leading to abnormal histology [16].

An analogous picture is emerging in a number of rare and well-differentiated neoplastic forms, with *gsp* affecting cellular organization of permissive mature tissues. Acting early during tumor progression, *gsp* prevents the correct maturation of cell precursors possibly unbalancing intracellular signaling relevant to cell maturation. The list of *gsp* tumors dramatically extended in the last few years (Table 2) covering virtually all regions of the gastroenteric tract, from gastric to colorectal mucosa including accessory organs such as pancreas and liver.

As for bone and pituitary, the mutation is likely underlining a signaling pathway pivoting around cAMP (see below) that is sufficient to distort tissue differentiation or maintenance but only exceptionally to guide full malignant transformation.

The first report to identify *gsp* in the digestive tract describes a screen for biomarkers discriminating among pancreatic cysts [17]. These lesions are pockets filled with fluids. Quite common with aging, cysts are usually revealed by radiology exams. Cysts pose a serious clinical challenge, as occasionally they may be cancerous and justify surgical intervention. Yet, the final diagnosis can only be made by histological exam of the resected organ. *Gsp* was identified as a sensitive and specific marker of intraductal papillary mucinous neoplasm (IPMN). Although it remains debated if IPMN should be considered a direct precursor of pancreatic ductal adenocarcinoma (PDA), the treatment of choice is resection since patients with IPMN are at high risk to develop PDA [18].

*Gsp* is present in low grade IPMN and does not increase with the level of the dysplasia [19]. Discriminating IPMN in two subgroups, namely mucinous/intestinal and tubular subtypes, Hosoda et al. frequently found *gsp* in the first group, considered more indolent [20]. Tamura et al. reported analogous findings although no correlation was found between GNAS status and IPMN histologic grade or clinical characteristics, including patient postoperative outcomes [21].

IPMN substantially lacks significant symptoms. Nonetheless, there is a latent but significant impact of *gsp* and cAMP signaling in neoplastic transformation of selected microenviroments. Chronic inflammation is often characterized by *gsp+* lesions. In pancreas, neoplastic lesions including IPMN are typically associated to chronic inflammation and fibrosis. Gastric foveolar metaplasia in the duodenum was considered a reactive process caused by inflammatory conditions before a genetic component was suggested by the higher *gsp* prevalence in transformed areas as compared to the surrounding healthy tissue. A correlation with the inflammatory process is observed in liver, where *gsp* was reported to define a rare subgroup of inflammatory tumors characterized by STAT3 activation mediated by GNAS directly upregulating target genes of the inflammatory IL-6-STAT3 pathway via Src [22].

Another puzzling link with inflammation is *gsp* incidence within a spectrum of somatic mutations (TP53, KRAS, SMAD4, CDKN2A, MLL3 and RNF43) shared by pancreatic tumors and cholangiocarcinoma developing in chronic inflammation induced by the parasite Opisthorchis viverrini [23]. Upregulated PKA synergizes with Wnt/β catenin to promote the slow progression of the tumor. However, the more aggressive outcome with *Opisthorchis* infection might be stimulated by the exogenous etiology of the inflammatory process.

Elevated Gαs activity can be accomplished by multiple mechanisms, or its consequences achieved by other activators on the same pathway [24]. For instance, in pituitary, *gsp* negative somatotroph tumors show loss of methylation and biallelic GNAS expression, that is likely to translate in increased wt Gαs expression [25, 26]. Cortisol-producing adenomas, another *gsp*+ tumor (Table 2), shows that alterations of components of Gαs downstream signaling pathway, namely a defective form of the regulatory subunit of PKA [27], could provide a surrogate of *gsp* activity in preventing normal cell maturation and tissue organization. Screens for somatic mutations in cortisol-producing adenomas demonstrated mutually exclusive mutations activating PKA and Gαs [28].

A better understanding of the pathogenesis triggered by *gsp* would likely provide important diagnostic tools to preoperatively predict the histological subtype for pancreatic lesions [29] and possibly others.

### Functional consequences of *gsp* signaling

**Fig. 3:**
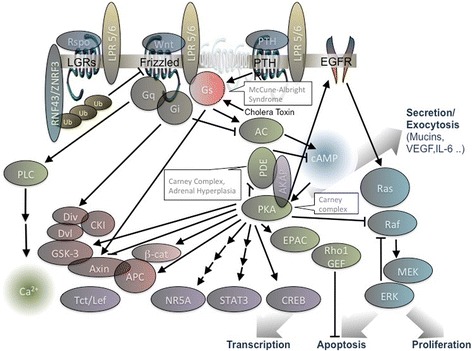
GPCR signaling is functional to almost any aspect of cell physiology including the organization of the stem cells niche. Gs coupled receptors like LGR and PTHR cooperate with FZD producing very articulated downstream signalling that is also modulated by single transmembrane domain co-receptors. Here a highly simplified scheme representing signalling intermediates described in the text. Multiple arrows indicate indirect activation. In the callout, congenital diseases associated to mutations upregulating the downstream pathway

The classical signaling pathway portrayed downstream of Gαs depicts the activation of adenylyl cyclase and a consequent increase in cytosolic cAMP (Fig. 3). In turn, cAMP interacts with the regulatory subunits of PKA setting the catalytic subunits free to phosphorylate multiple targets. Consistently, PKA is up-regulated in *gsp*+ neoplasms such as IPMN [30] or appendiceal adenoma [31]. But PKA activation may not be the sole target of *gsp* signaling, as PKA mutations are not commonly reported in sporadic cancers of the GI tract. At least in tissues where *gsp+* commonly occurs, this could suggest that *gsp* activates additional targets in parallel to PKA. On the other hand, the very rare Carney complex (CC, a heterozygous, autosomal dominant syndrome caused by mutations up-regulating PKA in all tissues), partially overlaps MAS symptoms including adrenocortical, pituitary, thyroid, skin tumors and pigmented lesions, myxomas (combined with FD symptoms is defined Mazabraud's syndrome), schwannomas, liver cancer and even IPMN [32]. Widespread PKA activation in all cells may phenocopy more focal lesions that contin a *gsp+* mutation within susceptible tissue.

In cortical cells renewing adrenal cortex, GNAS and PKA mutations produce benign lesions that lead to Cushing disease. Nonetheless, there are some distinctions: *gsp* produces micronodular disease in MAS or ACTH-independent macronodular hyperplasia, and, occasionally cortisol producing adenoma; PKA mutations produce primary pigmented nodular adrenocortical disease [33].

The molecular consequences of the upregulation of the Gs->cAMP->PKA axe on different tissue microenvironments remains to be clarified. Essential targets for activation in *gsp* neoplasias have not yet been identified but candidates include PKA and its substrates, such as AKAP and other scaffolding proteins, ion channels, receptor tyrosine kinases, and cAMP response element-binding (CREB) protein that drives transcription of cAMP-responsive element-containing genes [34, 35], PKA functional and structural interaction with the EGF receptor [36] could be particularly relevant. Gαs could promote cell proliferation by PKA-dependent cross-talk with the EGF pathway at multiple levels, but in particular at the level of KRAS. KRAS is one of the most frequently mutated genes in human tumors [37] and its simultaneous expression with *gsp* is found in certain tumor types.

Thyroid carcinoma express activating mutations of Gαs (12.5%), most commonly with activating mutations of the KRAS-paralog NRAS (8.5%). The co-occurrence of mutations simultaneously activating Gαs and KRAS is not rare in tumors like IPMN but may be coincidental, perhaps resulting from contamination of samples with cells from two independent origins.

However, in other tumor types, like pituitary [38] or muscle [37, 39], activating mutations of Ras family members are extremely rare and associated with malignant features, likely representing a late event in tumor progression. In any case, activating alleles of GNAS certainly occur in cancer independently of activating alleles of KRAS (Table 1).

Although synergy between GNAS and KRAS is not obvious, multiple studies analyzed the simultaneous presence of both oncogenes. Whereas some cases found a positive correlation [40, 41], others observed different frequencies of mutations affecting KRAS and GNAS as in colloid vs. tubular subtypes of invasive IPMN. This may suggest two separate progression pathways [19] supporting previous findings in papillary neoplasms of the bile duct [42, 43].

Cooperative signaling between Gαs and Kras proteins was recently demonstrated in a mouse model of IPMN. Conditional expression of gsp increased intracellular cAMP concentration and fibrosis, but mice developed IPMN-like lesions, with PKA activation and mucin over-production, as in human, only when mutant KRAS was co-expressed. [44] *Gsp* may provide a selective advantage to cell precursors that, instead of undergoing normal differentiation, become an indolent neoplasm usually described by a relatively rare and fine-focused diagnosis showing precise histologic characteristics. Retrospective analysis reported hepatobiliary and pancreatic neoplasms in about 30% of MAS patients [45–47]. Cell autonomous signals produced by the activation of both pathways are likely to converge as cAMP controls MAPK signaling at multiple levels.

Field effect (i.e. the existence of histologically abnormal microfoci within apparently normal tissue that predisposes to the occurrence of simultaneous and independent primary tumors) [43, 48] may also contribute to disease progression. For example, *gsp* is present in about half of IPMN samples but TCGA identified activating alleles of GNAS with wild type KRAS in only 3% of PDA. By contrast, about half of IPMN samples and upwards of 92% of PDA have activating alleles of KRAS but wild type GNAS (Table 1). This suggests *gsp* may contribute to chronic pancreatic disease but secondary mutations, such as KRAS activation, are necessary for transformation and tumor progression [43, 49]. Mosaic analysis is required to determine whether cell autonomous signaling or field effect explain the apparent interaction between Kras and Gαs proteins.

### Tissue and cell specificity

Although Gαs is ubiquitously expressed, *gsp* only produces significant consequences in “permissive organs” [1, 50]. As mentioned in the first paragraph, in almost all endocrine tissues GNAS is transcribed from the maternal allele [51], therefore a predominance of *gsp* vs WT GNAS caused by tissue specific imprinting could partially explain why penetrance is so low elsewhere.

In adult pituitary, Gαs is monoallelically expressed from the maternal allele and indeed Hayward et al. identified the mutation in the maternal allele in 21 out of 22 GH-secreting adenoma analyzed. Yet, imprinting was relaxed for GNAS while still fully in place for the other genes belonging to the same locus (NESP55 and XLαs) [52]. In bone, the paternal allele is not differentially methylated and both alleles are expressed as in other pluripotent stem cells [9]. Imprinting by itself is therefore insufficient to explain tissue specificity.

Even in the same tissue, *gsp* oncogenic effects can be highly lineage cell specific and prevent, rather than promote, proliferation/survival. [53] In pituitary, the mutation emerges as a proliferatory stimulus only in GH-secreting cells while in other pituitary cells derived from common progenitors (gonadotroph or lactotroph derived) cAMP accumulation inhibits growth [26].

In the skin, *gsp* has never been associated with neoplastic growth. On the contrary, in an animal model of basal-cell carcinogenesis the Gs–PKA axis was recently shown to play an important tumor suppressive role. By controlling s-HH and Hippo signaling PKA defined the size of the stem cell compartment limiting self-renewal. Upregulating its signaling by exogenous *gsp* expression caused hair follicle stem cells exhaustion [54]. Consistently, in MAS the presence of *gsp* does not promote melanoma but only causes hyperpigmentation of melanocytes by upregulating tyrosinase gene [55].

Several issues should be addressed before we understand the basis of such tissue- and cell-specificity. Among them, cell lineage specific expression of Gαs effector isoforms (that include 10 adenylyl cyclases isoforms, 2 PKA catalytic subunits [26]) or the mechanisms opposing the increased cAMP levels (i.e. 11 subfamilies of phosphodiesterases [56]).

Tissue specific expression of different transcripts and alternative compartmentalization of Gαs may also differentiate the response by restricting signaling at precise subcellular locations [57, 58]. Traditionally, heterotrimeric G protein signaling has been described only at the plasma membrane. Acylated Gαs is anchored to the plasma membrane, but the post-translational modification is reversible and Gαs is also found cytosolic, particularly in the constitutively active form. [59] Recently, it became clear that upon GPCR activation, internalized receptors produce intracellular signaling by upregulating adenylyl cyclase located in the endosomal compartment [60, 61].

**Fig. 4:**
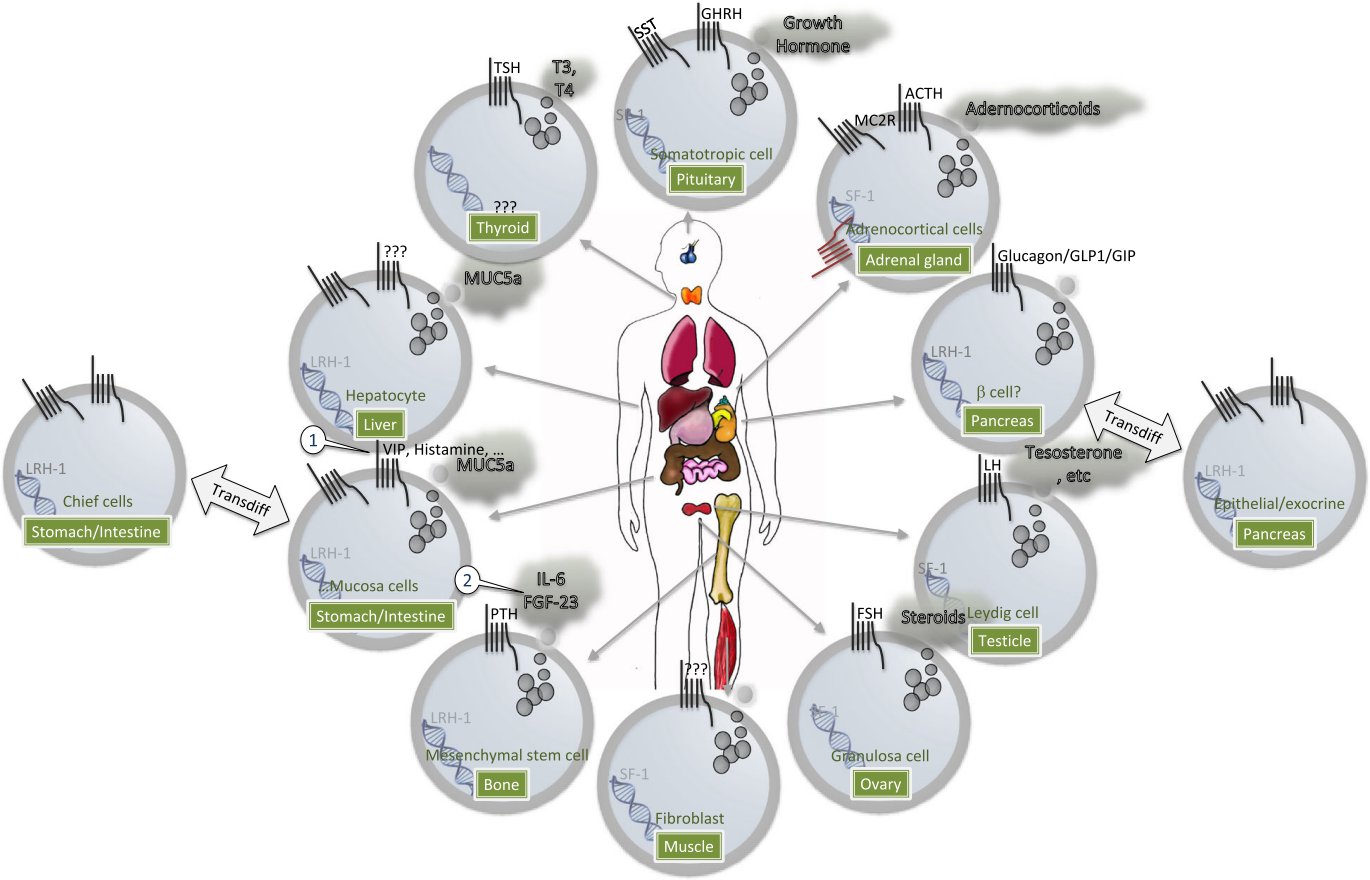
GPCR dependent production of intracellular cAMP determines secretory function in specialized cells of different exocrine organs. Among GPCRs determinant for the differentiation and function of tissues displaying the *gsp*+ phenotype, are secretin family receptors (glucagon, GIP, secretin, VIP, GHRH) and LGR receptors (LGR 1-8). The same pathways instrumental to zonation and differentiation are also likely acting on transcriptional programs related to the differentiation stage reached by the cells. NR5A master regulators are present in cells displaying the *gsp+* phenotype (thyroid [95], osteoblast [93], somatotropic, pancreatic cells [98], hepatocytes, intestinal crypt [91], adrenocortical, Leydig and granulosa cells [99])

cAMP is a diffusible signaling molecule but is concentrated in local microdomains due to the action of phosphodiesterases [62]. Another potential mechanism to determine “cell specific” responses comes from the subcellular localization of PKA [33] mediated by AKAP that scaffolds several components of the pathway mentioned above [63] (Fig. 3). The list could continue, but summarizing, the expression of a constitutively active mutant is expected to affect the signaling network at multiple districts and unlikely to entirely mimic the stimulus of a Gs coupled receptor, nor to produce a generalized cAMP increase. The implications on cell physiology are unpredictable but may explain the different phenotypes observed in MAS vs Carney complex and the absence of phenotype in most tissues of the organism. More work is required to identify the critical steps in neoplastic transformation, beginning with the most relevant Gs mediated pathways in each tissue specific cell type (Fig. 4).

### A GPCR perspective of the niche microenvironment

The analysis of GNAS in neoplasias of the gastrointestinal tract is shedding new light on the significance of the mutation. Gastric adenocarcinoma and most gastric tumors are *gsp* negative (Table 2). Conversely, gastric neoplasia of fundic gland and pyloric gland adenoma are *gsp+*. Both subtypes are rare and share neck cell/chief cell lineage phenotype [64]. In the normal gastric mucosa, the digestive-enzymes secreting chief cells differentiate from mucous neck cells via trans-differentiation [65]. Neoplastic cells possess characteristics of immature chief cells transitioning from mucous neck cells to serous chief cells. Probably, the same diversion from advanced phases of the differentiation program occurs in the intestine where a potential histogenic link is observed between these gastric lesions and analogous lesions appearing as gastric foveolar epithelium but commonly present in duodenal biopsy specimens [66]. Depending on the absence or presence of oxyntic glands, these lesions are classified into gastric foveolar metaplasia or gastric heterotopia. Microdissection-based analysis of three gastric heterotopia lesions identified common GNAS mutations in both components of the gastric mucosa: foveolar epithelium and oxyntic glands suggesting that the initial mutation occurred in stem cells, before they differentiated into both epithelial components and that the mutation did not cause detectable morphological changes in oxyntic glands [66]. This scenario is reminiscent of osteoblasts maturation described above.

In pituitary, *gsp* emerges only in somatotrophs that can also transdifferentiate from closely-related secretory cells. The emerging picture is that GNAS activating mutations allow cell survival only after a specific commitment has been undertaken and confer growth advantage only to precise lineages.

Stem cell self-renewal is critical in embryos and in adult to repopulate tissues undergoing continuous renovation. Cross-talk from multiple ligands evoking Gs-signaling orchestrates self-renewal: Wnt, sHH and R-spondins being among the best-described examples.

GPCR signaling is also involved in the differentiation of immature progenitors during cell migration. Leucine-rich repeat-containing G-protein coupled receptors (LGRs) regulate maturation of adult stem cells during renewal of papillary intestine mucosa or the patterning of hepatic lobules. In other tissues analogous processes coordinately regulate maturation, migration, and proliferation. For instance, adrenal cortex undergoes constant cell renewal as proliferation in the outer cortex continues with centripetal cell migration and differentiation according to cell location along the cortex medulla axis. Angiotensin II and ACTH signaling are crucial for modulating the size of each zone [67].

The same GPCR acting during development or in adult stem cell niches may also regulate the terminally differentiated cell. Examples include ACTH signaling to MC2R in zona fasciculata differentiation of adrenal cortex, PTH in osteoblasts, and LH gonadotropin in Leydig cells. In these cells, trophic hormones evoke Gαs-cAMP dependent growth and hormone release. Many exocrine or endocrine organs are among those affected by constitutively active Gs protein (see Table 2 and Fig. 4). Consistent with the clinical features presented by affected patients [68], glyco-proteins are secreted in IPMN [21, 42], IPNN of the bile duct [42], duodenum [66], appendix [69], cervical mucosa [70] by glands trans-differentiated towards a gastric phenotype, and hormones are secreted in pituitary (GH), thyroid (TSH), and gonads (LH and FSH).

Hence, multiple examples support the idea that *gsp* may bypass GPCR mediated signals to subvert differentiation and promote proliferation in progenitor cells rather than terminally differentiated cells.

### cAMP and Wnt signaling pathways

Wnt has been implicated in the oncogenesis of all “permissive tissues” of the *gsp* mutation (thyroid [71], bone [72], pituitary [73], stomach [74], intestine [75], colon, pancreas, adrenocortical [76]). In adrenal cortex, gene expression analysis aimed at understanding cAMP tumorigenic activity indicated cell cycle and Wnt signaling as the most affected pathways [33].

Concerted cAMP and Wnt signaling is likely to represent a hallmark of *gsp+* neoplasias. Cross-talk could occur at any level of the signaling cascade. Wnt activates intracellular signaling by simultaneously interacting with two co-receptors: a lipoprotein receptor-related protein (LRP5/6) and one out of ten members of the Frizzled family (FZD1-10) characterized by conserved cysteine-rich domain and seven transmembrane domains. In addition to coupling Gs and other heterotrimeric G proteins, FZDs interact with the scaffolding protein disheveled. [77] As a result, FZDs act upstream of three principal signaling mechanisms: the ‘canonical’, “planar cell polarity” and ‘calcium’ pathways [78].

Interestingly, in addition to FZDs**,** several classical GPCRs interact with LRP6 and activate downstream signaling. These include prostaglandin E2 and F2, M1 acetylcholine muscarinic, lysophosphatidic acid, gonadotrophin-releasing hormone and PTH type 1 (PTH1R) receptors.

In the canonical pathway, FZDs activates β-catenin by disassembly of an intracellular inhibitory complex formed by GSK3, adenomatosis polyposis coli (APC), axin and casein kinase Iα (CKIα). Wnt stimulation prevents the formation of the complex, cytoplasmic β-catenin is stabilized, and translocates to the nucleus to associate with transcription factors.

Hashimoto et al. analyzed a cohort of 20 patients affected by familial adenomatous polyposis with inherited APC mutations. Out of 6 cases with associated pyloric gland adenomas of the stomach, 5 were *gsp+*, all carrying APC mutations and high nuclear β-catenin expression levels [79]. All other patients and other types of lesions (foveolar adenoma and fundic gland polyps) were *gsp* negative.

The cooperative effect of *gsp* and APC inactivation was also demonstrated in animal models of intestinal tumor formation [75]. A correlation between the two pathways is supported by animal models in which increased PKA activity led to high prostaglandin E2 levels and activated Wnt signaling [33, 80].

In colon cancer without functional APC, cell proliferation is stimulated by the proinflammatory metabolite prostaglandine E2, an agonist for the Gs-coupled EP2. Under these circumstances, Gαs-GTP, activated either by the ligand or by mutation, directly interacts with the RGS homology (RH) domain of axin. The interaction sets GSK-3β free from the complex and leads to the stabilization and nuclear translocation of β-catenin [81]. Analogously, PTH1R was shown to promote the direct association of Gαs-GTP with axin [82].

Other critical points of convergence of the two pathways may involve less well characterized signaling intermediates. An emerging finding shared by ovarian development and digestive mucosa or adrenal cortex regeneration mediated by adult stem cells [83], is the involvement of a protein complex module featuring (Fig. 3):

- Wnt binding to FZD and LRPs
- a family of four cysteine rich proteins R-spondins (Rspo1-4) binding to LGR 4, 5 and 6
- E3 ubiquitin ligases ZNRF3 and RNF43.

RNF43 and the closely related homolog ZNFR43 act as co-receptors to transduce signaling across the plasma membrane, they target cytosolic loops of FZD promoting its ubiquitination, internalization and degradation [84]. Mutations inactivating RNF43/ZNRF43 are expected to reduce FZD ubiquitination and to upregulate Wnt signaling. Similar genetic anomalies are observed in approximately 90% of colorectal cancers as well as in other cancer types, such as hepatocellular carcinomas or gastric cancers [33] including most *gsp+* neoplasias: liver fluke associate cholangiocarcinoma, IPMN, ovary [85], adrenocortical carcinomas [80, 86]. In addition to mutations, epigenetics or other regulatory mechanisms reducing RNF43 expression could play an analogous effect synergizing with GNAS in the formation of IPMN and other neoplasia in the pancreas [87].

PKA was shown to phosphorylate and upregulate β-catenin signaling [88, 89] possibly in conjunction with other transcription factors. In adrenal tumors autonomously producing cortisol, gain of function mutations in β-catenin and in either Gαs or PKA were reported as mutually exclusive [90]. Other transcription factors regulated by cAMP are two nuclear receptors binding to the same consensus sequences and named steroidogenic factor 1 (SF-1/NR5A1) and liver receptor homologue 1 (LRH-1/NR5A2).

NR5A2 has an important role in the gastrointestinal system regulating functions such as bile acid and pancreatic fluid biosynthesis and secretion, glucose sensing and cell renewal in the crypt. The latter aspect is mediated by the interaction with CREB and β-catenin [91]. In addition to being expressed in the intestinal crypt cells [92], NR5A2 is also expressed by osteoblasts [93]. NR5A1 expression profile is restricted to adrenal cortex, gonads, spleen, pituitary, gonadotropes, hypothalamic VMN [94]. NR5A1 target genes are implicated at every level of the hypothalamic-pituitary-gonadal axis and gonadal or adrenal steroidognesis [94].

An interplay between Wnt and ACTH->cAMP->PKA pathways within the process of renewal and lineage conversion has been suggested in zona fasciculata development of the adrenal cortex. The mechanism portrays PKA phosphorylation of NR5A1. A subtle balance between Wnt and PKA activation determines functional zonation titrating NR5A1 and β-catenin. By this means, Wnt and Adrenocorticotropric Hormone (ACTH) stimulation determine and maintain cortex renewal. [67]

NR5A1 regulation by cAMP is also reported to direct functional differentiation of thyroid progenitor cells and to be involved in adenoma development [95]. NR5A2 was shown to play a role in intestine tumorigenesis controlling enterocytes cell cycle and inflammatory cytokines. NRA5A2 is a susceptibility locus for human pancreatic cancer. Intriguingly, in an animal model of KRAS driven neoplasia, NRA5A2 function constrains tumor initiation [96].

By compromising the correct tuning of Wnt signaling, *gsp* would thus distort the cellular response to surrounding signals. However, since an optimal level of Wnt/βcatenin signaling is essential to tumor formation [97], a constitutive activation of Gαs may not necessarily allow full transformation [43] explaining why the oncogenic effect is manifest only under specific circumstances in permissive tissues.

In summary, loss-of-function mutations in the Wnt signalosome that inhibit β-catenin may synergize with *gsp,* suggesting that.constitutive Gαs activity lowers Wnt signaling activation threshold.

## Conclusions

*Gsp* is emerging as an oncogene acting in multifactorial transformation processes in low-grade or benign neoplasia. In the digestive tract, it is often associated with papillary morphology and high mucin secretion, reminiscent of previously described endocrine tumors. High Gαs activity may interfere with signaling in immature stages but is not sufficient to progress to invasive carcinoma. Therefore, *gsp* could be a marker for differential diagnosis of early neoplasia.

## Abbreviations

ACTH: Adrenocorticotropric hormone
APC: Adenomatosis polyposis coli
CKIα: Casein kinase Iα
CREB: cAMP response element-binding
FD: Fibrous dysplasia
GH: Growth hormone
GHRH: hypothalamic hypophysiotropic hormone/GR Releasing hormone
IPMN: Intraductal papillary mucinous neoplasia
LRG: Leucine-rich repeat-containing G-protein coupled receptors
LRH-1/NR5A2: Liver receptor homologue 1
MAS: McCune Albright Syndrome
PDA: Pancreatic ductal adenocarcinoma
SF-1/NR5A1: Steroidogenic factor 1
